# Primary headache disorders susceptibility is associated with fasting-related headache: evidence from a multinational study across 14 countries

**DOI:** 10.3389/fneur.2026.1859536

**Published:** 2026-06-25

**Authors:** M. Ceren Akgor, K. Saghır, A. İ. Can, F. G. Sahbaz, E. U. Özcelik, A. C. Atalar, N. Ergin, M. O. Orun, A. A. Madani, E. A. A. Ibrahim, M. Togha, S. Amirguliyev, Y. Alabwah, J. A. Hashel, I. M. Dardar, R. E. Sahli, S. B. Sassi, M. Nada, N. Shalaby, Y. Dehal, Y. Naji, M. Amine, K. Alfred, R. Karacı, E. Yanık, G. Gürsoy, H. T. Celik, S. Tasdelen, E. Ekizoğlu, E. K. Orhan, D. Agırcan, H. Genc, T. Gesoglu Demir, F. M. Domac, N. Köse, N. Tepe, S. Öztürk, E. K. Cengiz, K. Erdogan, İ. Yıldırım, B. R. Hasırcı Bayır, E. A. Demirel, R. Ghouri, B. Tasdelen, H. Bolay, A. Ozge, N. Kissani

**Affiliations:** 1 Department of Neurology, University of Health Sciences Ankara Atatürk Sanatorium Training and Research Hospital, Ankara, Türkiye; 2 Neuroscience and Neurotechnology Center of Excellence (NÖROM), Ankara, Türkiye; 3 Department of Neurology and Clinical Neurophysiology, Helios University Hospital Wuppertal, Wuppertal, Germany; 4 Faculty of Health, Witten/Herdecke University, Witten, Germany; 5 Prof. Dr. Aynur Özge Headache Clinic, Mersin, Türkiye; 6 Department of Neurology, Izmir University of Economics Medical Point Hospital, İzmir, Türkiye; 7 Department of Neurology, School of Medicine, Acıbadem University, Istanbul, Türkiye; 8 Department of Neurology, İstanbul Physical Therapy and Rehabilitation Training and Research Hospital, İstanbul, Türkiye; 9 Department of Neurology, Faculty of Medicine, Pamukkale University, Denizli, Türkiye; 10 Department of Neurology, Van Training and Research Hospital, University of Health Sciences, Van, Türkiye; 11 Department of Neurology in Rashed Hospital, MBRU University, Dubai, United Arab Emirates; 12 Neurology Department, National Centre for Neurological Centre, Alneelain University, Khartoum, Sudan; 13 Headache Department, Iranian Center of Neurological Research, Neuroscience Institute, Tehran University of Medical Sciences, Tehran, Iran; 14 Headache Department, Neurology Ward, Sina Hospital, Medical School, Tehran University of Medical Sciences, Tehran, Iran; 15 Department of Neurology and Clinical Neurophysiology, Azerbaijan State Institute of Advanced Training of Doctors Named after Aziz Aliyev, Baku, Azerbaijan; 16 Sabah Al Salem University City, Kuwait City, Kuwait; 17 Department of Neurology, Department of Medicine, Ibn Sina Hospital, Faculty of Medicine, Kuwait Univesity, Safat, Kuwait; 18 Department of Neurology, National Police Medical and Surgical Center, Djibouti City, Djibouti; 19 Department of Neurology, College of Medicine Benghazi University, Benghazi, Libya; 20 Department of Neurology, National Institute Mongi Ben Hamida of Neurology, Tunis, Tunisia; 21 Neurology Department, Cairo University, Giza, Egypt; 22 Hospitalier de Specialite de Nouakchott, Nouakchott, Mauritania; 23 Neurology Department, Agadir University Hospital, Agadir, Morocco; 24 Neurology Department, Mohammed V Military Hospital, Rabat, Morocco; 25 Department of Neurology, Yaounde Central Hospital, Yaounde, Cameroon, Faculty of Medicine and Biomedical Sciences, University of Yaounde I, Yaounde, Cameroon; 26 Department of Neurology, Erenkoy Mental and Nervous Diseases Training and Research Hospital, University of Health Sciences, Istanbul, Türkiye; 27 Department of Algology, University of Trakya, Edirne, Türkiye; 28 Department of Neurology, Umraniye Training and Research Hospital, Istanbul, Türkiye; 29 Department of Neurology, Haydarpasa Numune Education and Research Hospital, Istanbul, Türkiye; 30 Department of Neurology, Faculty of Medicine, Istanbul University, Istanbul, Türkiye; 31 Department of Neurology, Faculty of Medicine, Harran University, Şanlıurfa, Türkiye; 32 Gaziantep City Hospital, Health Application and Research Center, Neurology Clinic, Gaziantep, Türkiye; 33 Department of Physical Therapy and Rehabilitation, Faculty of Physical Therapy and Rehabilitation, Hacettepe University, Ankara, Türkiye; 34 Department of Neurology, Faculty of Medicine, Balıkesir University, Balıkesir, Türkiye; 35 Department of Neurology, Gaziantep Islamic Science and Technology University, Gaziantep, Türkiye; 36 Department of Neurology, Balıkesir University Health Practice and Research Hospital, Balıkesir, Türkiye; 37 Department of Neurology, Kütahya University of Health Sciences, Kütahya, Türkiye; 38 Department of Neurology, Faculty of Medicine, Bulent Ecevit University, Zonguldak, Türkiye; 39 Department of Neurology, Faculty of Medicine, Mersin University, Mersin, Türkiye; 40 Department of Biostatistics and Medical Informatics, School of Medicine, Mersin University, Mersin, Türkiye; 41 Department of Neurology and Algology, Faculty of Medicine, Gazi University, Ankara, Türkiye; 42 Neuropsychiatry Center, Gazi University, Ankara, Türkiye; 43 Laboratory of Clinical and Experimental Neuroscience, Faculty of Medicine, Cadi Ayyad University, Marrakech, Morocco; 44 Department of Neurology, Bengrir UM6P University, Ben Guerir, Morocco

**Keywords:** fasting-related headache, migraine, primer headache disorders, Ramadan fasting, risk stratification

## Abstract

**Background:**

Fasting-related headache has traditionally been attributed to dehydration, caffeine withdrawal, and sleep disruption, but pre-existing primary headache disorders may also play an important role in determining susceptibility.

**Objectives:**

To determine the prevalence, clinical characteristics, and predictors of headache during Ramadan fasting and to explore the relative contribution of pre-existing primary headache disorders and the lifestyle-related factors assessed in this study to headache occurrence during fasting.

**Methods:**

In this multinational cross-sectional survey across 14 countries, adults (18–65 years) observing Ramadan fasting completed a 17-item questionnaire. Headache during Ramadan was the primary outcome. Classification and regression tree (CRT) modelling were used to identify predictors and characterize fasting related headache.

**Results:**

Headache attacks occurred mainly in the afternoon/pre-iftar period and improved after iftar in 54.6% of cases. Pre-existing primary headache disorders, particularly migraine, were more strongly associated with headache occurrence within the exploratory CRT model than the lifestyle variables assessed in this study.

**Conclusion:**

Fasting-related headache is highly prevalent and is influenced by both lifestyle-related factors and underlying headache susceptibility. A history of primary headache disorders was strongly associated with headache occurrence during Ramadan fasting, highlighting the importance of considering individual headache history alongside fasting- related exposures when assessing vulnerability.

## Introduction

Fasting-related headache is traditionally conceptualized as a secondary headache driven primarily by lifestyle-related factors such as dehydration, caffeine withdrawal, and sleep disruption. Lifestyle- related factors are frequently linked to fasting- related headache, but they do not entirely explain why headache occurs in some fasting individuals and not in others. Individual differences are susceptibility may also be relevant. Ramadan offers an opportunity to examine these relationships under relatively uniform fasting conditions. However, previous studies have been geographically restricted, methodologically diverse and largely focused on potential triggers rather than individual predisposition. In particular, the independent contribution of pre-existing primary headache disorders, especially migraine, has not been adequately quantified in previous research.

They are also not allowed to smoke, take oral medication, receive intravenous fluids, or receive nutrients during Ramadan (1). As the Islamic Calendar is lunar, the duration of fasting varies across years and geographical regions, ranging from approximately 12 h in New Zealand to up to 18 h in Greenland (2).

During Ramadan, Muslims experience significant lifestyle modifications that may play a role in headache development, notably alterations in sleep patterns. Early awakening for a pre-dawn meal before fasting (suhoor) and prayers, together with late-night wakefulness, shortens sleep duration. Additionally, substantial changes occur in meal timing, dietary intake, and fluid consumption, with fasting individuals typically consuming two main meals, suhoor and evening meal for breaking the fast (iftar) (3, 4). In ICHD-3, “headache attributed to fasting” is classified as a secondary headache attributed to a disorder of homeostasis (5). The diagnostic criteria for the fasting headache in the ICHD-3 are a diffuse, non-pulsating headache, usually mild to moderate, occurring during and caused by fasting for at least 8 h, and relieved after eating. The ICHD-3 definition may not adequately reflect the clinical diversity of headache attacks that occur in the context of long-term, repetitive fasting, such as during Ramadan. As a large proportion of the global population observes Ramadan annually, understanding the epidemiology and determinants of fasting-related headache attacks is relevant not only to clinical care but also to broader population health.

Emerging evidence suggests that fasting may act less as a primary cause of headache and more as a trigger that unmasks an underlying susceptibility, particularly in individuals with a sensitized trigeminovascular system. This raises the possibility that fasting-related headache may reflect differences in individual susceptibility to headache, in addition to fasting-associated lifestyle exposures.

The observation that headache develops in some fasting individuals but not in others suggests that fasting-related exposures may not fully explain headache risk. Individual susceptibility may also play a role. We therefore conducted a large multinational study to investigate the prevalence, characteristics, and predictors of headache during Ramadan fasting. Additionally, to explore the relative contribution of pre-existing headache disorders and the lifestyle-related variables assessed in this study in relation to headache occurrence during Ramadan fasting.

This study aimed to explore whether pre-existing headache disorders may contribute to fasting-related headache beyond the lifestyle-related variables assessed in the present survey.

## Patients and methods

This cross-sectional, multilingual survey investigated fasting-related headache among adults observing Ramadan across 14 countries. A structured 17-item questionnaire collected information on demographic characteristics, lifestyle factors, headache history, and fasting- related headache features. The questionnaire was developed using a symptom-oriented framework aligned with the International Classification of Headache Disorders, 3rd edition (ICHD-3) to facilitate clinically meaningful headache classification in a population based setting. Variables included age, sex, country of residence, smoking status, habitual coffee or tea consumption, history of primary headache disorders, headache occurrence during previous Ramadan periods, headache intensity, duration, location, associated symptoms, timing, response to breaking the fast, and acute medication use. Participants reporting a history of chronic headache were asked to indicate the headache type from predefined categories, including migraine, tension type headache, cluster headache and trigeminal neuralgia. Participants were recruited through a hybrid approach involving both physician-assisted data collection in clinical settings and direct self-completion via a secure online platform. Items assessing headache timing, localization, and relief after breaking the fast required a single response, whereas associated symptoms and selected headache characteristics allowed multiple responses. Missing responses were excluded on a variable-by-variable basis, and analyses were performed using available data for each variable. Physicians were instructed to clarity of questionnaire items when necessary to enhance data quality and consistency across sites. This dual recruitment strategy was implemented to maximize reach while preserving clinical interpretability across heterogeneous populations. Eligible participants were adults aged 18–65 years who observed Ramadan fasting. Of the 2,433 survey respondents, 2,358 fulfilled the predefined eligibility criteria and were therefore included in the final analyses. The multinational design encompassed participants from 14 countries, increasing external validity and allowing assessment of fasting-related headache across diverse environmental and cultural contexts.

Data from 2,358 participants across 14 countries, mainly from Türkiye, Tunisia, Libya, Morocco, Kuwait, and the UAE, were included in the final analyses. The study was conducted during the holy month of Ramadan. We collected data from March 22 to April 22, 2023. Statistical analyses were performed using SPSS version 23.0. Descriptive statistics were used to summarise baseline characteristics and headache features. In addition, an exploratory CRT analysis was performed to identify risk profiles and interactions among predictors. Model complexity and validation was controlled using pruning and split-sample validation. Pruning is an optimization technique to avoid overfitting by removing unnecessary branches and nodes of a decision tree. The post pruning strategy was used; the tree was grown until stopping criteria which were 5 for maximum tree depth and minimum number of case for 50 for child nodes were met. Then, the tree was trimmed automatically to the smallest subtree based on the specified maximum difference in risk. The default risk value (1) was used in the modelling. Other stopping criteria is to reduce impurity of tree. The classification and regression tree (CRT) method aims to maximize within-node homogeneity and create splits that reduce impurity. Impurity indicates that a node does not represent a homogenous subset of cases.

Because our dependent variable was nominal, Twoing algorithm was used to obtain the best split of two categories. The minimum decrease in impurity was measured using minimum change in improvement. The default was 0.0001. To assess how well the structure of decision tree can be generalized to a larger population, split-sample validation was used. The data was splitted into training (60%) and test (40%) sets. Training Set was used to build the actual structure of the decision tree and test set was used to evaluate the model. The model’s prediction accuracy was evaluated both training and test sets separately. The value was assumed as moderate for 0.60–0.70, good for 0.70–0.90 and perfect for >0.90.

The following candidate predictors were entered into the CRT: age, sex, smoking, habitual caffeine intake, and history of primary headache disorders.

## Results

### General characteristics of participants

A total of 2,358 participants from 14 countries were included in the final analysis, providing a large and geographically diverse cohort of fasting individuals. The primary outcome, defined as the occurrence of headache during the most recent Ramadan, was reported by 1,616 participants (68.5%). As a secondary outcome, 1760 participants (74.6%; 95% CI 72.1–75.6) reported experiencing headache while fasting during Ramadan 2023.

The secondary analyses further examined the characteristics of headache experienced during fasting in Ramadan 2023, including their timing, localization, and associated symptoms, and response after breaking the fast. The female to male ratio was approximately 2:1 and 68.0% of participants were aged 30 years or older. Half of the cohort was from Türkiye, followed by Tunisia (7.4%), Libya (6.3), and Morocco (6.2). Regarding lifestyle habits, 17.6% of participants were smokers, and 62.8% reported routinely starting the day with a cup of coffee or tea. Baseline sociodemographic and lifestyle characteristics are summarized in Table 1.

**Table 1:** Baseline sociodemographic and lifestyle characteristics of participants.

Characteristic	*n* (%)
Gender	
Male	755 (32.0)
Female	1,603 (68.0)
Age	
≤30 years	792 (33.6)
>30 years	1,566 (66.4)
Country of origin	
Türkiye	1,271 (53.9)
Tunisia	175 (7.4)
Libya	148 (6.3)
Morocco	147 (6.2)
Kuwait	130 (5.5)
United Arab Emirates	108 (4.6)
Other	379 (16.1)
Smoking	
Yes	415 (17.6)
No	1943 (82.4)
Starts the day with coffee or tea	
Yes	1,482 (62.8)
No	876(37.2)

### Characteristics of headache during Ramadan

**Figure 1:**
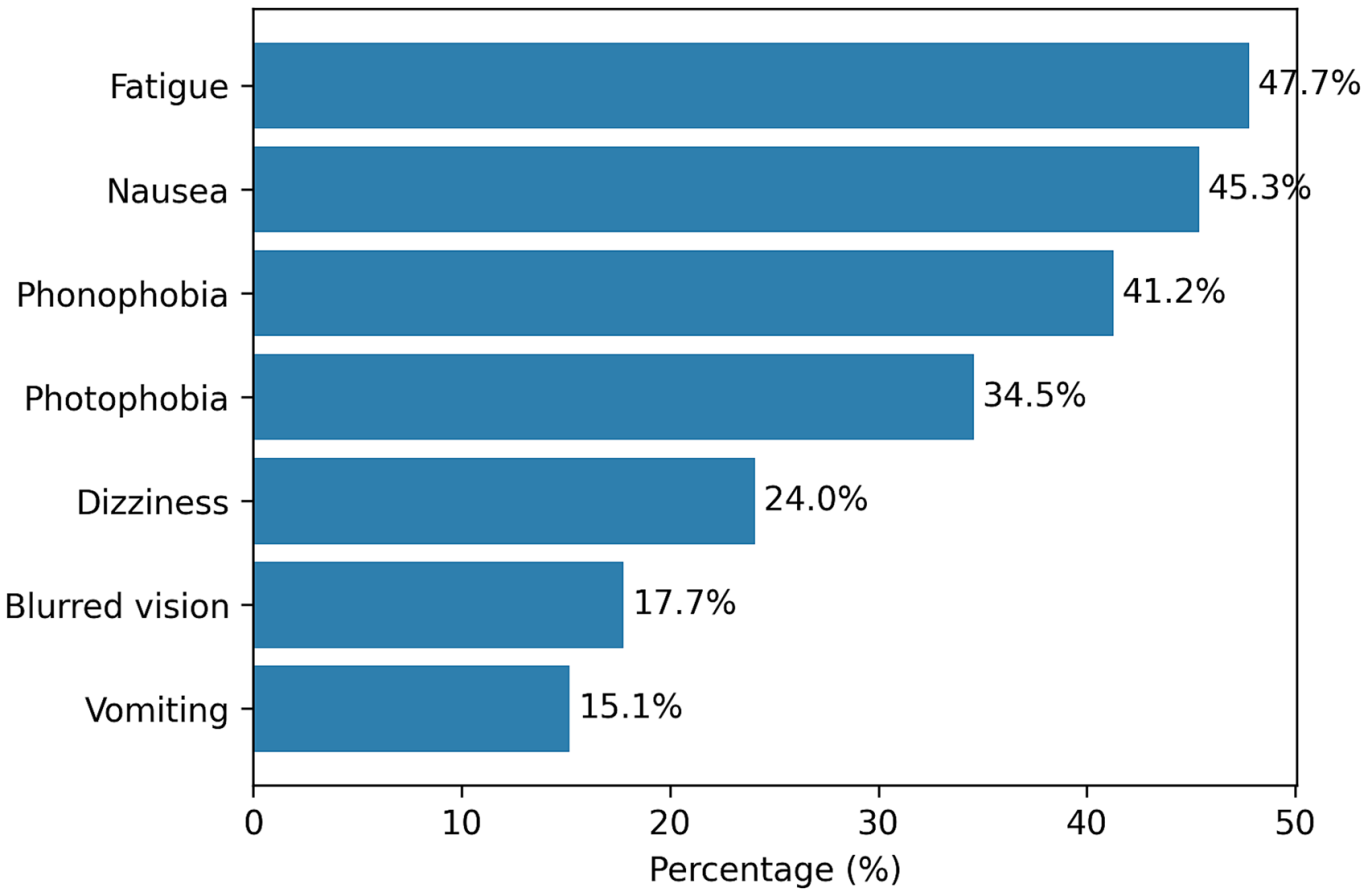
Distribution of associated symptoms reported with fasting-related headache during Ramadan.

Among the 1760 participants who reported headache while fasting during Ramadan 2023 (74.6%). As shown in Table 2, headache attacks were most common during the afternoon and pre-iftar periods, suggesting a temporal relationship with cumulative fasting-related physiological stressors. Pain was of moderate intensity (mean NRS 6.5 ± 1.7) and exhibited both tension-like (55.7%) and pulsatile (53.3%) features. These findings support the concept that fasting-related headache represents a spectrum of phenotypes rather than a uniform condition. More than half of participants reported headache episodes lasting longer than 2 h, while fatigue (47.7%), nausea (45.3%), phonophobia (41.2%), photophobia (34.5%) and the most frequently reported associated symptoms (Figure 1). Additional headache characteristics are summarized in Table 2.

**Table 2:** Clinical characteristics of fasting-related headache during Ramadan 2023.

Characteristic	*n* (%)
Headache phenotype^*^	
Heaviness or tension	955 (55.7)
Pulsation	914 (53.3)
Tingling	148 (8.6)
Relief after breaking the fast^†^	
Yes	936 (54.6)
No	777 (45.4)
Timing of onset^*^	
Morning	355 (20.5)
Midday	405 (23.4)
Afternoon	653 (37.8)
Evening before breaking fast (pre-iftar period)	648 (37.5)
Evening after breaking fast (post-iftar period)	380 (22.0)
Pain location^*^	
Forehead	695 (43.4)
Bilateral	484 (30.2)
Right side	405 (25.3)
Back of the head	363 (22.7)
Left side	363 (22.7)
Vertex	6 (0.5)
Associated symptoms^*^	
Fatigue	843 (47.7)
Nausea	801 (45.3)
Phonophobia	728 (41.2)
Photophobia	609 (34.5)
Dizziness	424 (24.0)
Blurred vision	312 (17.7)
Vomiting	266 (15.1)

Values are *n* (%), percentages are calculated among participants reporting headache while fasting during Ramadan 2023 with available responses for each item; denominators therefore vary across items.

^*^Multiple responses permitted. ^†^Relief assessed after iftar.

### Medication for a fasting headache

Overall 80.6% reported acute medication use, with over-the-counter pain relievers being the most frequently used. The most commonly used medications were paracetamol (62.7%), naproxen (28.3%), and ibuprofen (19.5%), whereas triptans were used relatively infrequently. Mean headache severity did not differ between medication users and non-users (7.08 vs. 7.00; *p* = 0.95), and medication use was not associated with a history of migraine or headache intensity.

However, information regarding medication timing, dosage, frequency, treatment indication, and perceived response was not collected; therefore, treatment effectiveness could not be evaluated.

### Associated factors with headache during Ramadan

To further explore factors associated with headache occurrence during the most recent Ramadan, CRT model was constructed using headache occurrence during the most recent Ramadan as the dependent variable. The dataset was randomly divided into training (60%) and test (40%) subsets. The model achieved an overall classification accuracy of 70.7% in the training set and 69.2% in the test set, indicating moderate predictive performance.

Variable importance analysis identified pre-existing chronic headache as the strongest contributor to classification (normalized importance: 100%), pulsatile headache characteristics (37.6%), coffee or tea consumption (27.8%), tension-type headache features (22.4%), smoking status (21.4%), and age (16.0%). The CRT model identified clinically interpretable risk profiles and demonstrated hierarchical interactions among headache history, age, and the lifestyle variables included in the survey (Table 3). Within the exploratory CRT framework, pre-existing headache disorders consistently demonstrated higher relative importance than the lifestyle variables included in the present survey. The CRT analysis identified clinically interpretable subgroup patterns associated with varying probabilities of headache occurrence during Ramadan. Among the headache-related variables included in the exploratory CRT model, migraine subtype showed relatively high variable importance values.

**Table 3:** Variable importance values derived from the CRT model predicting headache occurrence during the most recent Ramadan.

Rank	Variable	Importance	Normalized importance (%)
1	Chronic headache history	0.024	100.0
2	Migraine type	0.015	64.2
3	Pulsatile headache characteristics	0.009	37.6
4	Coffee/tea consumption	0.007	27.8
5	Tension-type headache characteristics	0.005	22.4
6	Smoking status	0.005	21.4
7	Age	0.004	16.0
8	Headache severity (NRS)	0.003	13.8
9	Nausea	0.002	10.2
10	Cluster headache	0.001	4.6
11	Duration of headache	0.001	4.2
12	Trigeminal Neuralgia	0.001	3.8
13	Tingling	0.001	3.6
14	Dizziness	0.001	3.4
15	Osmophobia	0.001	2.7
16	Phonofobia	0.001	2.6
17	Photofobia	0.001	2.1
18	Compressive pain	<0.001	0.9
19	Vomiting	<0.001	0.3

Variable importance values were obtained from the Classification and Regression Tree (CRT) model. Normalized importance values are expressed relative to the most influential predictor (chronic headache history = 100%).

Variable importance values were obtained from the Classification and Regression Tree (CRT) model. Normalized importance values are expressed relative to the most influential predictor (chronic headache history = 100%).

At the root node, 68.6% of participants reported headache during the Ramadan preceding the survey. The first split was based on the presence of a pre-existing primary headache disorder, with participants reporting such a history showing a markedly higher prevalence of headache during the previous Ramadan.

**Figure 2:**
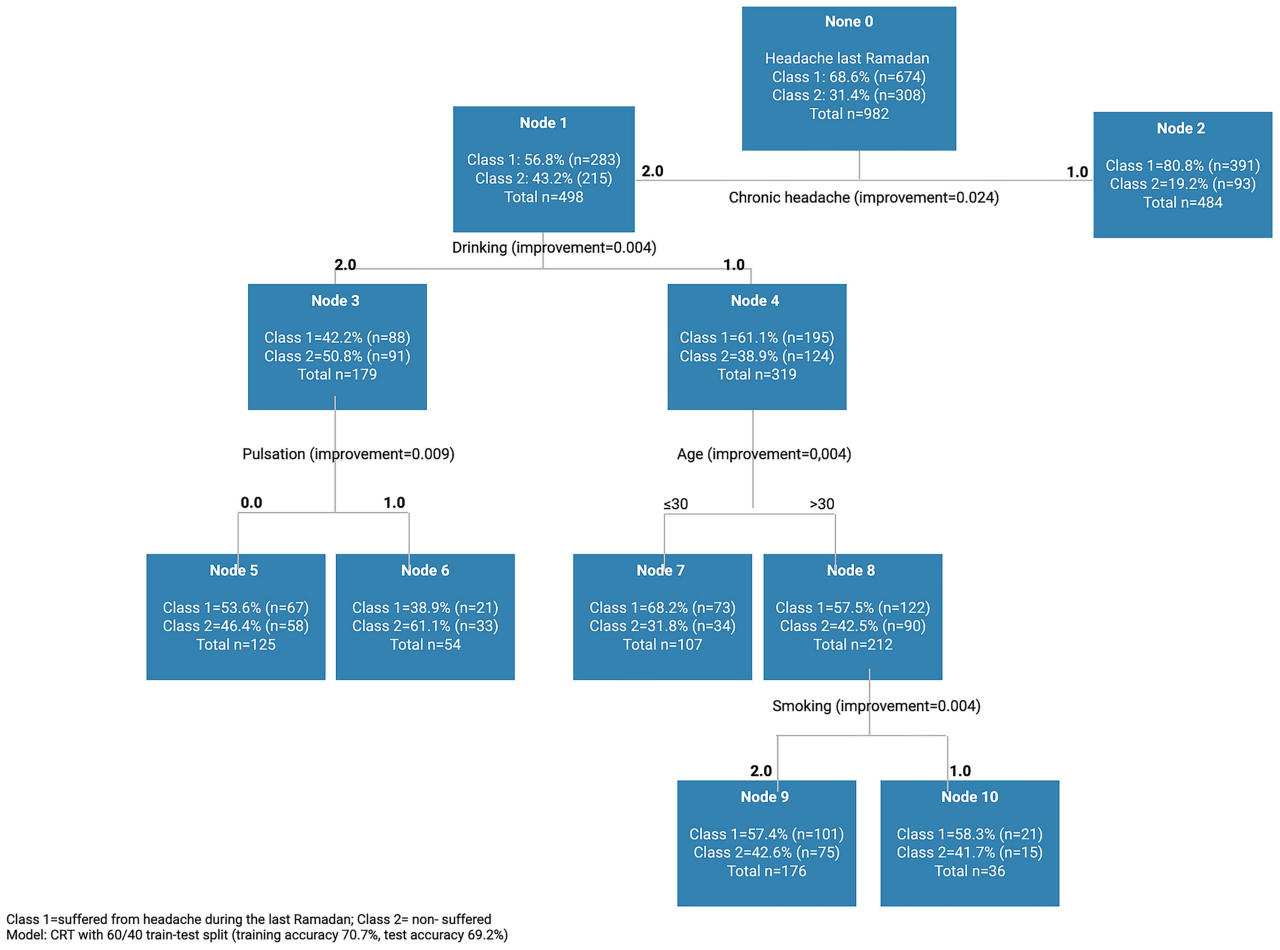
CRT analysis illustrating hierarchical predictors of headache occurrence during the most recent Ramadan. The tree structure demonstrates the sequential contribution of clinical and demographic variables to headache risk stratification. The model achieved classification accuracies of 70.7 and 69.2% in the training and test datasets, respectively. This figure is created with Biorender.

Subsequent splits further stratified participants into subgroups with varying probabilities of experiencing headache attacks, revealing heterogeneity in risk profiles across the cohort. The probability of headache ranged from 38.9 to 80.8% across terminal nodes. Among participants without a history of primary headache disorder, younger age and habitual coffee or tea consumption contributed to further risk stratification within the CRT model. Smoking contributed only limited additional discrimination between risk groups (Figure 2).

These findings suggest that a prior history of primary headache disorder was more strongly associated with headache occurrence during Ramadan within the exploratory CRT model than the lifestyle variables assessed in this study. Within the CRT framework, chronic headache history represented the first partitioning variable in the decision tree.

## Discussion

This large-scale multinational study challenges the conventional view that fasting-related headache is primarily driven by lifestyle-related factors. Rather than pointing to a single cause, our findings suggest that individuals with pre-existing primary headache disorders, particularly migraine, may be more vulnerable to developing headache during Ramadan. This interpretation aligns with the current understanding of migraine as a disorder of altered sensory processing and homeostatic vulnerability. While fasting has traditionally been conceptualized as a direct cause of headache through mechanisms such as dehydration, hypoglycemia, caffeine withdrawal, and sleep disruption (1–4), our results suggest that these factors are better understood as modulators acting on an underlying individual vulnerability.

Although lifestyle-related factors such as caffeine withdrawal, sleep disruption, and dehydration have been implicated in fasting-related headache (1–4), their relative contribution could not be directly assessed in the present study. This may partly explain the inconsistent findings reported across smaller or geographically restricted studies (3, 6–8). In contrast, pre-existing primary headache disorders, particularly migraine, emerged as the strongest predictor in our analyses. Rather than supporting a simple distinction between “lifestyle-related” and “biological” mechanisms, these findings align with current models of migraine, in which headache attacks arise from the interaction between external triggers and individual susceptibility. In this context, fasting-related changes in hydration, sleep, caffeine intake, and metabolic state may increase the likelihood of headache in susceptible individuals.

An important consideration is that our study examined headaches occurring during fasting rather than establishing a diagnosis of headache attributed to fasting according to ICHD-3 criteria. The high frequency of pulsatile pain, nausea, photophobia, and phonophobia, together with the observation that only about half of participants reported improvement after iftar, suggests that many reported attacks may have represented primary headache attacks—particularly migraine—triggered by fasting. Accordingly, our findings should be interpreted as describing headache occurrence during fasting rather than confirming headache attributed to fasting in all affected participants.

The observed mixed headache phenotype, combining tension-like and pulsatile characteristics, further supports the notion that fasting-related headache represents a heterogeneous spectrum rather than a single diagnostic entity. This spectrum likely includes both non-specific homeostasis-related headache and fasting-triggered exacerbations of underlying migraine. The high prevalence of associated symptoms such as fatigue, nausea, and phonophobia further suggests that the burden of fasting-related headache extends beyond pain intensity alone and reflects broader neurobiological dysregulation during fasting.

The high rate of acute medication use, primarily with paracetamol and NSAIDs, coupled with the absence of association between medication use and headache severity, suggests a potential mismatch between treatment practices and effectiveness in real-world fasting conditions. This may be explained by delayed or suboptimal timing of medication intake, particularly given that many individuals defer treatment until after iftar or take medication at suhoor, potentially missing the optimal therapeutic window as physiological stress accumulates later in the fasting period (9). In contrast, previous randomised controlled trials have demonstrated the effectiveness of prophylactic strategies, such as pre-fasting administration of long-acting COX-2 inhibitors, in reducing fasting-related headache (4, 10, 11). These findings highlight a potential gap between evidence and clinical practice and underscore the importance of anticipatory management strategies.

These results have important clinical implications. Individuals with a history of migraine or chronic headache should be considered a high-risk group prior to Ramadan. Targeted pre-Ramadan counselling, including optimization of preventive treatment, regulation of sleep patterns, and gradual adjustment of caffeine intake, may reduce the burden of fasting-related headache. A risk-stratified approach focusing on vulnerable individuals may be more effective than uniform lifestyle recommendations applied across all fasting populations.

Our findings suggest that fasting- related headache is unlikely to be explained by a single mechanism. Changes commonly experienced during Ramadan, including reduced fluid intake, altered sleep schedules, fluctuations in caffeine consumption, and metabolic changes, may all contribute to headache occurrence. At the same time, the effect of these factors is unlikely to be uniform across individuals. In our cohort, participants with a history of primary headache disorders were consistently more likely to report headache during fasting. Rather than arguing against a role for lifestyle-related factors, this finding suggests that such exposures may have a greater impact in individuals who are already predisposed to headache. Viewed in this way, fasting- related headache is best understood as the result of fasting- associated physiological changes acting on a background of individual susceptibility (12, 13).

The CRT findings should be interpreted with caution, given the method’s exploratory nature and the absence of conventional multivariable effect estimates. The primary purpose of the analysis was to identify clinically interpretable subgroup patterns and hierarchical associations that may inform future hypothesis-driven studies.

Several limitations should be acknowledged. First, the cross-sectional design precludes causal inference; therefore, observed associations should be interpreted with caution. Second, data were collected through a self-administered online survey, introducing the possibility of both recall bias and self-selection bias, as individuals experiencing headache symptoms may have been more likely to participate. In addition, headache diagnoses were not clinically confirmed but were based on self- reported history and symptom profiles, although a structured symptom- based approach aligned with ICHD-3 criteria was used to improve classification and interpretability (5). The study also lacked objective measurements of fasting- related lifestyle and physiological factors, including hydration status, caffeine consumption, sleep parameters, and metabolic changes. Consequently, the relative contribution of these factors may not have been fully captured. Furthermore, participants from Türkiye represented more than half of the cohort, and women accounted for approximately two-thirds of respondents, which may limit the generalizability of the findings. Because the survey was disseminated through multiple online channels, the total number of individuals who received the invitation was unknown, precluding calculation of a formal response rate; no weighting procedures were applied. In addition, the exploratory CRT approach used in this study does not provide adjusted effect estimates or establish independent causal relationships among predictors. The identified associations and hierarchical variable structure should therefore be interpreted cautiously and viewed primarily as hypothesis-generating.

Despite these limitations, the large sample size and multinational recruitment across 14 countries strengthen the external validity of the findings and support their relevance across diverse cultural and geographical settings. Future prospective, diary- based studies incorporating objective assessments of hydration, metabolic status, sleep, and circadian rhythms are warranted to better characterize temporal relationships and clarify the mechanisms underlying fasting- related headache (1, 14).

In conclusion, fasting-related headache is highly prevalent during Ramadan. Both fasting- related exposures and underlying headache susceptibility likely contribute to its occurrence, with individuals with pre-existing primary headache disorders appearing particularly vulnerable. These findings may help inform risk stratification and more targeted preventive approaches in fasting populations.

Previous studies conducted during Ramadan and other religious fasting periods, such as Yom Kippur, have also reported an increased occurrence of headache during fasting, although these studies were generally limited by smaller sample sizes and more restricted populations (15, 16).

## Data Availability

The raw data supporting the conclusions of this article will be made available by the authors, without undue reservation.
